# Is prior cancer history a hindrance for non-small cell lung cancer patients to participate in clinical trials?

**DOI:** 10.1186/s12885-023-10551-9

**Published:** 2023-02-15

**Authors:** Jing-Sheng Cai, Yun Li, Xun Wang

**Affiliations:** 1 Department of Thoracic Surgery, Peking University People’s Hospital, Beijing, 100044 People’s Republic of China; 2 Thoracic Oncology Institute, Peking University People’s Hospital, Beijing, 100044 People’s Republic of China

**Keywords:** Prior cancer, Non-small cell lung cancer, Survival

## Abstract

**Background:**

This study aimed to explore the effect of a prior cancer history on the survivals of resected non-small cell lung cancer (NSCLC) patients.

**Methods:**

Kaplan–Meier method with a log-rank test was used to compare overall survival (OS) and disease-free survival (DFS) between groups. Propensity score matching (PSM) method was used to reduce bias. The least absolute shrinkage and selection operator (LASSO)-penalized Cox multivariable analysis was used to identify the prognostic factors.

**Results:**

A total of 4,102 eligible cases were included in this study. The rate of patients with a prior cancer was 8.2% (338/4,102). Patients with a prior cancer tended to be younger and have early-stage tumors when compared with those without prior cancer. Before PSM, the survivals of the patients with a prior cancer were similar to those of the patients without prior cancer (OS: *P* = 0.591; DFS: *P* = 0.847). After PSM, patients with a prior cancer and those without prior cancer still had comparable survival rates (OS: *P* = 0.126; DFS: *P* = 0.054). The LASSO-penalized multivariable Cox analysis further confirmed that a prior cancer history was not a prognostic factor for both OS and DFS.

**Conclusions:**

A prior cancer history was not associated with resected NSCLC patients’ survivals, and we proposed that it might be reasonable for clinical trials to enroll the NSCLC patients with a prior cancer.

**Supplementary Information:**

The online version contains supplementary material available at 10.1186/s12885-023-10551-9.

## Background

With the development of novel treatment modalities, cancer patients’ survivals are improved, which contributes to a growing number of cancer survivors [1]. Therefore, patients with a prior cancer are not rare. Lung cancer is still the leading cause of cancer-related mortality worldwide [2, 3]. It is estimated that one in five lung cancer patients has a prior cancer [1, 4].

It is a longstanding practice that the non-small cell lung cancer (NSCLC) patients with a prior cancer are excluded from clinical trials [5, 6]. The researchers deemed that previous malignancies and treatments may have an adverse impact on the survivals of this population subset [7], and thus, interfere with study outcomes. However, little evidence has been found to support this notion. Whether this exclusion criterion is evidence-based remains unclear [8]. In the real-world setting, this criterion has prevented many potential candidates from participating in clinical trials which might improve their survival outcomes [9, 10]. In addition, this criterion could halt the pace and generalizability of clinical trials [11, 12]. So, there is a pressing need to revisit this so-called eligible criterion and explore the exact role that it plays in NSCLC patients’ survivals.

In such instances, the current study analyzed the data of resected NSCLC patients from a large Chinese cohort to determine the effect of a prior cancer on the prognosis of NSCLC patients. We aimed to answer the question that whether NSCLC patients with a prior cancer should be excluded from clinical trials.

## Methods

### Patient selection

This is a retrospective review of a series of 7,931 consecutive resected patients in a single center. The database used in this study was reported before [13]. The inclusion criteria were that: [1] pathologically confirmed as primary NSCLC; and [2] received surgery. Patients were excluded when meeting the following criteria: [1] age < 18 years; [2] received neoadjuvant therapy; [3] N3 category; [4] M1 category; [5] positive surgical margin; [6] adenocarcinoma in situ; and [7] unavailable information. Then, the eligible patients were assigned to two groups: with a prior cancer group [prior cancer ( +)] and without prior cancer group [prior cancer (-)] according to the medical history recorded in the electronic system.

### Ethic

The Ethics Committee of Peking University People’s Hospital approved this study with the approved number: 2020 PHB 421–02. The informed consent was waived off by the Ethics Committee of Peking University People’s Hospital due to the retrospective design of the study.

### Perioperative treatments

In general, routine preoperative evaluations including history taking, physical examinations, hematological examinations, chest computed tomography (CT), brain magnetic resonance imaging, and cardiac and pulmonary function tests were performed. Positron emission tomography (PET) imaging was not mandatory in our center because it has not been covered by medical insurance in mainland China. The surgical approach and surgical extent were discussed and decided at a multidisciplinary team meeting. All included patients received completely resection. After surgery, the formalin-fixed paraffin-embedded tissue sections with hematoxylin and eosin staining were reviewed by the pathologists of our institution. Adjuvant therapies, usually four cycles of platinum-based doublet chemotherapies, were administered to the stage IIA-IIIB NSCLC patients according to the National Comprehensive Cancer Network (NCCN) guidelines [14] in this period.

### Follow-up and endpoints

In routine, postoperative follow-up was performed every three months for the first two years, every six months for the next three to five years and annually thereafter [13]. Chest CT, serum tumor markers and physical examinations were performed at each follow-up visit. When suspected of relapse, PET or brain MRI and bone scan were performed. Follow-up information was obtained through telephone calls and hospital visits. Overall survival (OS) and disease-free survival (DFS) were the primary endpoints of this study. OS was described as the period between the date of surgery and the date of death or the last known contact. DFS was described as the period between the date of surgery to the date of first recurrence or death.

### Statistically analysis

The non-normally distributed continuous variables were presented as median (range), which were compared using the Mann–Whitney U test. Categorical variables were presented as percentages, which were compared using Pearson Chi–square test or Fisher’s exact test. In the comparisons of four grid table data, when the expected frequency of the data is less than 1 or the total number of cases is less than 40, the Fisher´s exact test is performed. Survival outcomes were analyzed using the Kaplan–Meier method with a log–rank test. Propensity score matching (PSM) method was carried out to reduce the bias caused by the baseline confounders using the R package “MatchIt” [15]. A least absolute shrinkage and selection operator (LASSO) model was performed to select the potential prognostic variables using the R package “glmnet” [16]. The LASSO-selected variables were further entered into a forward stepwise multivariable Cox analysis to determine the final independent prognostic factors. The 8^th^ version of the tumor-node-metastasis (TNM) staging system [17] was used in this study. Complete data analysis was conducted in this study. The statistical analyses were performed using the IBM SPSS Statistics (version 25.0, IBM Corp, Armonk, NY, USA) and the R version 4.1.1 (The R Foundation for Statistical Computing, Vienna, Austria; http://www.r-project.org). Two-sided *P* < 0.05 was considered statistically significant.

## Results

### Baseline characteristic

A total of 4,102 eligible cases (with prior cancer: 338 cases; without prior cancer: 3,764 cases) were included. The detailed clinicopathological information is listed in Table 1. Regarding the entire cohort, the median age was 62 years (range: 18 to 86 years). Male and female were at a comparable proportion (50.4% vs. 49.6%). There were more younger patients (60 years vs. 61 years), females (66.9% vs. 48.0%) and non-smokers (74.6% vs. 64%) in the prior cancer ( +) group when compared with the prior cancer (-) group. Regarding the clinical tumor stage, patients in the prior cancer ( +) group had earlier T category and earlier N category tumors (T category: *P* = 0.007; N category: *P* = 0.048). Considering the surgical approach and surgical extent, more patients in the prior cancer (+) group received video-assisted thoracic surgery (VATS) and sublobectomy when compared with those in the prior cancer (-) group (surgical approach: *P* = 0.005; surgical extent: *P* = 0.011). Pathological findings demonstrated that patients in the prior cancer (+) group had more early-stage tumors (*P* = 0.004). Considering the patients in the prior cancer (+) group, there were 73 cases, 67 cases, 50 cases, 25 cases, 24 cases, 20 cases, 20 cases, 15 cases, 11 cases, 8 cases, 6 cases, 5 cases, 4 cases, 4 cases, 3 cases, 2 cases, and 1 case in the prior thyroid cancer, breast cancer, intestinal cancer, renal carcinoma, gastric cancer, uterus cancer, central nervous system tumor, ovarian cancer, bladder cancer, esophagus carcinoma, pancreatic carcinoma, prostatic cancer, NSCLC, Hodgkin lymphoma, nasopharyngeal carcinoma, liver cancer, and biliary tract cancer prior cancer group, respectively. Due to small number of cases in several groups, we combine the latter fourteen groups into one group: other.

**Table 1:** The baseline characteristics of the NSCLC patients with and without prior cancer

Characteristics	with prior cancer (*N* = 338)	without prior cancer (*N* = 3,764)	*P*
Age, years			< 0.001^a^
Median (range)	60 (29–84)	61 (18–86)	
Sex			< 0.001
Male	112 (33.1)	1,957 (52.0)	
Female	226 (66.9)	1,807 (48.0)	
Smoking			< 0.001
Non-smoker	252 (74.6)	2,410 (64.0)	
Smoker	86 (25.4)	1,354 (36.0)	
Family tumor history			0.023
Without	289 (85.5)	3,369 (89.5)	
With	49 (14.5)	395 (10.5)	
Preoperative comorbidity			0.120
Without	127 (37.6)	1,578 (41.9)	
With	211 (62.4)	2,186 (58.1)	
BMI			0.094^a^
Median (range)	24.1 (15.6–38.9)	24.0 (14.3–44.8)	
FEV1 (%)			0.470^a^
Median (range)	95.9 (21.1–259.0)	94.2 (52.6–165.0)	
DLCO (%)			0.003^a^
Median (range)	87.5 (11.6–160.8)	83.7 (41.5–708.0)	
Clinical T category			0.007
1	285 (84.3)	2,910 (77.3)	
2	44 (13.0)	589 (15.6)	
3	6 (1.8)	173 (4.6)	
4	3 (0.9)	92 (2.4)	
Clinical N category			0.048
0	309 (91.4)	3,268 (86.8)	
1	3 (0.9)	69 (1.8)	
2	26 (7.7)	427 (11.3)	
ASA grade			< 0.001^b^
1	36 (10.7)	758 (20.1)	
2	291 (86.1)	2,840 (75.5)	
3	11 (3.3)	162 (4.3)	
4	0 (0.0)	4 (0.1)	
Surgical approach			0.005
VATS	315 (93.2)	3,317 (88.1)	
Open	23 (6.8)	447 (11.9)	
Surgical extent			0.011
Lobectomy	230 (68.0)	2,769 (73.6)	
Sublobectomy	104 (30.8)	904 (24.0)	
Pneumonectomy	4 (1.2)	91 (2.4)	
Tumor location			0.521
Right upper lobe	119 (35.2)	1,193 (31.7)	
Right middle lobe	18 (5.3)	241 (6.4)	
Right lower lobe	61 (18.0)	784 (20.8)	
Left upper lobe	92 (27.2)	981 (26.1)	
Left lower lobe	48 (14.2)	565 (15.0)	
Histology			0.364
ADC	282 (83.4)	3,020 (80.2)	
SCC	45 (13.3)	596 (15.8)	
Other	11 (3.3)	148 (3.9)	
VPI			0.310
Without	271 (80.2)	2,928 (77.8)	
With	67 (19.8)	836 (22.2)	
LVI			0.062
Without	299 (88.5)	3,187 (84.7)	
With	39 (11.5)	577 (15.3)	
Tumor size, cm			0.005
Continue	1.8 (0.1–14.0)	1.5 (0.4–13.0)	
Pathologic T category			0.006
1	228 (67.5)	2,235 (59.4)	
2	93 (27.5)	1,165 (31.0)	
3	10 (3.0)	255 (6.8)	
4	7 (2.1)	109 (2.9)	
Pathologic N category			0.138
0	284 (84.0)	2,992 (79.5)	
1	21 (6.2)	302 (8.0)	
2	33 (9.8)	470 (12.5)	
Pathologic TNM stage			0.004
I	269 (79.6))	2,697 (71.7)	
II	26 (7.7)	486 (12.9)	
III	43 (12.7)	581 (15.4)	
Postoperative complications			0.142
Without	317 (93.8)	3,596 (95.5)	
With	21 (6.2)	168 (4.5)	
Adjuvant therapy			0.003
No	270 (79.9)	2,721 (72.3)	
Yes	68 (20.1)	1,043 (27.7)	
Hospital stay, day			0.668^a^
Continue	12 (2–61)	12 (2–99)	

*NSCLC* non-small cell lung cancer, *BMI* body mass index, *FEV1* forced expiratory volume in 1 s, *DLCO* diffusion capacity for carbon monoxide, *TNM* tumor-node-metastasis, *ASA* the American Society of Anesthesiologists, *VATS* video assisted thoracic surgery, *ADC* adenocarcinoma, *SCC* squamous cell carcinoma, *VPI* visceral pleural invasion, *LVI* lymphovascular invasion

^a^ Mann–Whitney U test

^b^ Fisher’s exact test

Fourteen variables, including age, sex, smoking, preoperative comorbidity, BMI, the American Society of Anesthesiologists (ASA) grade, surgical approach, surgical extent, histology, visceral pleural invasion (VPI), lymphovascular invasion (LVI), pathologic TNM stage, postoperative complication and adjuvant therapy, were entered into the PSM algorithm. After PSM, there were 338 well-matched pairs. The variables were all balanced well between these two groups (Table S1).

### Survival analysis

**Fig. 1:**
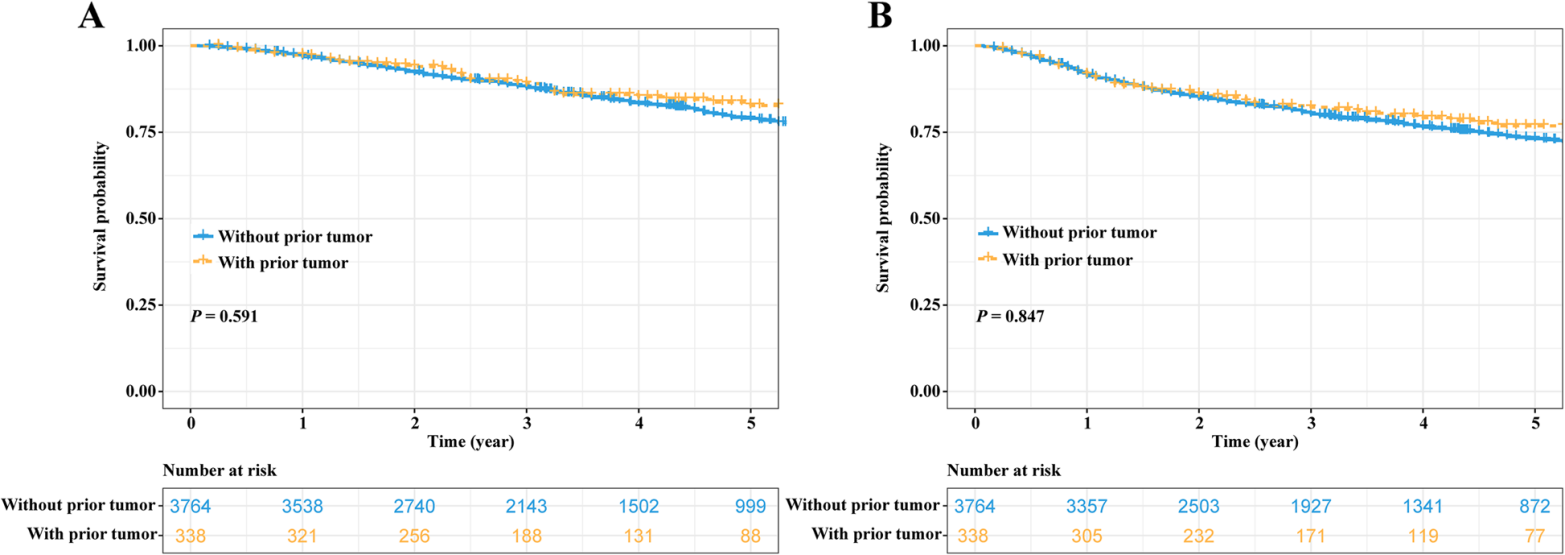
Survival comparisons between NSCLC patients with and without prior tumor before PSM. **A** OS comparison and **B** DFS comparison. NSCLC, non-small cell lung cancer; PSM, propensity score matching; OS, overall survival; DFS, disease-free survival

**Fig. 2:**
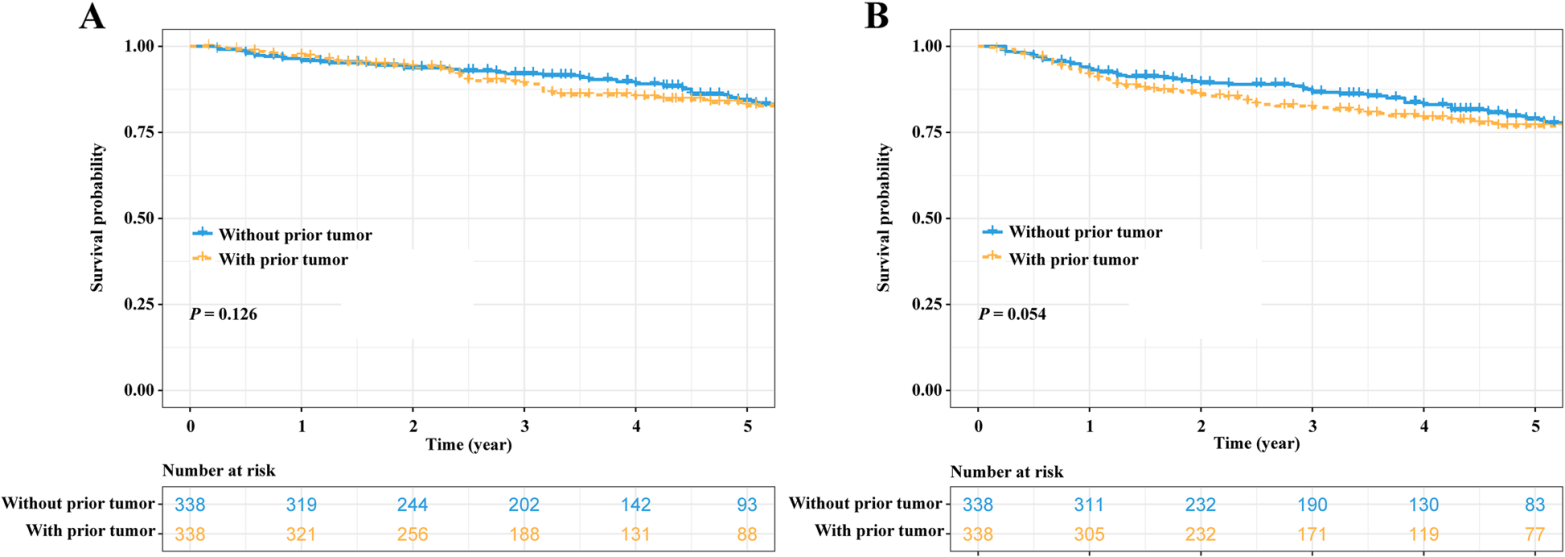
Survival comparisons between NSCLC patients with and without prior tumor after PSM. **A** OS comparison and **B** DFS comparison. NSCLC, non-small cell lung cancer; PSM, propensity score matching; OS, overall survival; DFS, disease-free survival

Before PSM, patients with and without prior cancer had comparable OS rates (5-year OS rate: 82.7% vs. 79.0%; *P* = 0.591; Fig. 1A) and DFS rates (5-year DFS rate: 76.8% vs. 73.1%; *P* = 0.847; Fig. 1B). In the matched cohort, patients in these two groups still had similar OS rate (5-year OS rate: 82.7% vs. 84.2%; *P* = 0.126; Fig. 2A). Regarding DFS, the DFS rates of the patients without prior cancer were better than those of the patients with prior cancer, but the difference was not statistically significant (5-year DFS rate: 78.9% vs. 76.8%; *P* = 0.054; Fig. 2B).

**Fig. 3:**
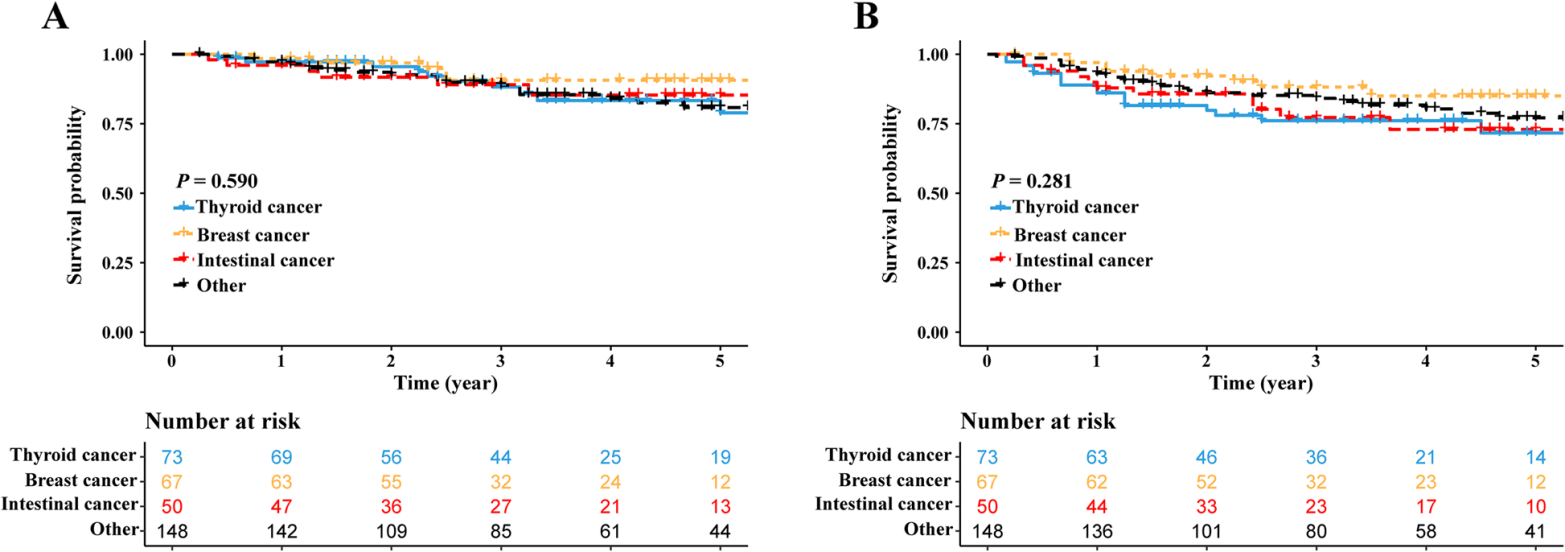
Survival comparisons stratified by the exact prior cancer types in the prior cancer ( +) cohort. **A** OS comparison and **B** DFS comparison. OS, overall survival; DFS, disease-free survival

In subgroup analysis, patients with prior cancer were divided into four subgroups (thyroid cancer, breast cancer, intestinal cancer and other) based on the exact prior cancer types. The results showed that although patients with prior breast cancer history seemed to have longer survivals, patients of these four subgroups had comparable survivals (OS: Fig. 3A; CSS: Fig. 3B).

### LASSO-penalized multivariable Cox analysis

A total of twenty-four variables, including age, sex, smoking, prior cancer history, family tumor history, preoperative comorbidity, hospital stay, body mass index (BMI), forced expiratory volume in 1 s (FEV1) %, diffusion capacity for carbon monoxide (DLCO) %, ASA grade, clinical T category, clinical N category, surgical approach, surgical extent, tumor location, histology, VPI, LVI, pathologic T category, pathological N category, pathological TNM stage, postoperative complication and adjuvant therapy, were entered into the LASSO model. Before PSM, the LASSO models demonstrated that age, BMI, histology, pathologic TNM stage and postoperative complication were the potential prognostic factors for OS, and histology, TNM stage and LVI were the potential prognostic factors for DFS. Multivariable Cox analysis further confirmed that age (*P* < 0.001), BMI (*P* < 0.001), histology (*P* < 0.001) and pathologic TNM stage (*P* < 0.001) were the independent prognostic factors for OS (Table 2). Regarding DFS, histology (*P* = 0.001), pathologic TNM stage (*P* < 0.001) and LVI (*P* < 0.001) were proven to be the independent prognostic factors (Table 2). After PSM, the results of the LASSO-penalized models showed that age (*P* = 0.001), histology (*P* = 0.013) and pathologic TNM stage (*P* < 0.001) were the independent prognostic factors for OS, and age (*P* = 0.001), histology (*P* = 0.034), pathologic TNM stage (*P* = 0.013), sex (*P* = 0.009), smoking (*P* = 0.019) and DLCO% (*P* = 0.026) were the independent prognostic factors for DFS (Table 3).

**Table 2:** LASSO-penalized multivariable Cox analysis before PSM

Characteristic	OS^a^			DFS^b^		
	HR	95% CI	*P*	HR	95% CI	*P*
Age, years			< 0.001			
Continue	1.042	1.0314–1.050				
BMI			< 0.001			
Continue	0.953	0.931–0.976				
Histology			< 0.001			0.001
ADC	1			1		
SCC	1.231	1.037–1.462		1.143	0.978–1.336	
Other	1.751	1.328–2.309		1.574	1.223–2.025	
Pathologic TNM stage			< 0.001			< 0.001
I	1			1		
II	3.110	2.533–3.819		3.248	2.703–3.904	
III	5.564	4.672–6.626		6.029	5.139–7.073	
Postoperative complication			0.292			
Without	1					
With	1.167	0.876–1.554				
LVI						< 0.001
Without				1		
With				1.335	1.138–1.567	

*LASSO* least absolute shrinkage and selection operator, *PSM* propensity score matching, *OS* overall survival, *DFS* disease-free survival, *BMI* body mass index, *ADC* adenocarcinoma, *SCC* squamous cell carcinoma, *TNM* tumor-node-metastasis, *LVI* lymphovascular invasion

^a^ Age, BMI, histology, pathologic TNM stage and postoperative complication were included in the multivariable Cox analysis of OS before PSM

^b^ histology, pathologic TNM stage and LVI were included in the multivariable Cox analysis of DFS before PSM

**Table 3:** LASSO-penalized multivariable Cox analysis after PSM

Characteristic	OS			DFS		
	HR	95% CI	*P*	HR	95% CI	*P*
Age, years			< 0.001			0.001
Continue	1.054	1.027–1.082		1.048	1.020–1.077	
BMI			0.258			0.418
Continue	0.965	0.907–1.027		0.972	0.908–1.041	
Histology			0.013			0.034
ADC	1			1		
SCC	1.509	0.912–2.498		1.474	0.786–2.767	
Other	2.597	1.329–5.076		2.671	1.261–5.655	
Pathologic TNM stage			< 0.001			0.013
I	1			1		
II	4.005	2.260–7.097		2.947	1.374–6.322	
III	5.343	3.284–8.695		2.999	1.007–8.933	
Postoperative complication			0.281			0.230
Without	1			1		
With	1.602	0.680–3.774		1.734	0.706–4.529	
Sex						0.009
Male				1		
Female				0.455	0.253–0.818	
Smoking						0.019
Non-smoker				1		
Smoker				2.213	1.139–4.300	
FEV1%						0.575
Continue				1.004	0.991–1.017	
DLCO%						0.026
Continue				0.982	0.967–0.998	
ASA grade						0.918
1				1		
2				1.258	0.648–2.440	
3				1.108	0.352–3.485	
4				1.652	0.933–4.237	
Surgical approach						0.190
VATS				1		
Open				1.527	0.811–2.875	
Tumor location						0.809
Right upper lobe				1		
Right middle lobe				0.640	0.225–1.823	
Right lower lobe				0.725	0.373–1.408	
Left upper lobe				0.781	0.450–1.356	
Left lower lobe				0.786	0.394–1.570	
VPI						0.874
Without				1		
With				1.042	0.625–1.738	
LVI						0.271
Without				1		
With				1.414	0.763–2.619	
Pathologic N category						0.454
0				1		
1				1.540	0.631–3.759	
2				1.823	0.603–5.512	

*LASSO* least absolute shrinkage and selection operator, *PSM* propensity score matching, *OS* overall survival, *DFS* disease-free survival, *BMI* body mass index, *ADC* adenocarcinoma, *SCC* squamous cell carcinoma, *TNM* tumor-node-metastasis, *FEV1* forced expiratory volume in 1 s, *DLCO* diffusion capacity for carbon monoxide, *ASA* the American Society of Anesthesiologists, *VATS* video assisted thoracic surgery, *VPI* visceral pleural invasion, *LVI* lymphovascular invasion

^a^ Age, BMI, histology, pathologic TNM stage and postoperative complication were included in the multivariable Cox analysis of OS after PSM

^b^ Age, BMI, histology, pathologic TNM stage, postoperative complication, sex, smoking, FEV1%, DLCO%, ASA grade, surgical approach, tumor location, VPI, LVI and N category were included in the multivariable Cox analysis of DFS after PSM

## Discussion

In this study, we investigated the effect of a prior cancer history on the survivals of resected NSCLC patients from a large Chinese cohort. The results showed that patients with and without prior cancer had similar survival rates both before and after PSM. The LASSO-penalized multivariable Cox analysis demonstrated that a prior cancer history was not a prognostic factor for survivals. Therefore, our data supported the notion that it might be unreasonable to exclude the NSCLC patients with a prior cancer history from clinical trials.

In our study, the incidence of patients with a prior cancer was 8.2%, which was lower than the previous studies [7, 8, 18, 19] where the authors reported that the frequency ranged from 15 to 20%, but was similar to Aguilo et al.’s study [20]. Our data also showed that patients in the prior cancer ( +) group were younger than those in the prior cancer (-) group. The reason behind the difference could be explained by the fact that patients with a prior cancer could be more engaged in screening program [7], which might contribute to early diagnosis of NSCLC. In addition, our finding that patients in the prior cancer ( +) group had more early-stage tumors when compared with the patients in the prior cancer (-) group also supported the assumption. The early-stage tumors allowed more patients in the prior cancer ( +) group received VATS and sublobectomy. To date, there are scant data to compare the baseline clinicopathological characteristics between these two group patients [6–8, 18–22]. Our study provided comprehensive knowledge of these patients to address this gap.

Several studies have attempted to identify the role that a prior cancer history plays in the survivals of NSCLC patients. In the study by Monalve et al. [7], the authors reviewed the data of 821,323 NSCLCs from the National Cancer Database (NCDB) and demonstrated that the OS of the patients with a prior cancer was better than those without cancer history (9.8% vs. 9.5%, *P* < 0.001). They further clarified that a prior cancer history posed a negative impact on patients with early-stage tumors but posed a favorable impact on those with advanced-stage tumors [7]. The strength of their study was the large data set of the NCDB. However, although subgroup analyses were conducted in their study, the baseline imbalance of groups might compromise their conclusions. In addition, the 6^th^ edition of TNM staging system was used in their study, which might lend inconvenience to clinical practice. Pruitt et al. [18] presented a large study of 42,910 early-stage lung cancer patients of whom 21% had a prior cancer from the Surveillance, Epidemiology and End Results (SEER) database. Their data showed that patients with and without prior cancer had similar survivals which was akin to our results. Nevertheless, DFS was not an endpoint of their study. It is known that many clinical trials select DFS as the primary endpoint. Therefore, lacking the data of DFS might limit clinical reference value. Laccetti et al. [8, 19] also explored the effect of a prior cancer history on the NSCLC patients’ survivals from the SEER database and obtained a similar conclusion that a prior cancer history had no effect on patients’ survivals. In our view, the data recorded in the public databases is rough, and other potentially important factors such as preoperative comorbidity, postoperative complications and novel pathologic findings (LVI and VPI) was not thoroughly explored, which might lead to an unreliable conclusion.

We conducted a relatively large study of 4,102 resected NSCLC patients. To the best of our knowledge, this is the first study using the PSM method to reduce the bias caused by the observed confounding variables in the baseline characteristics. Thus, our results were more reliable and persuasive. The patients included in this study received standard treatments. In most previous studies however, the inclusive criteria were not rigor, and patients who received no treatment were also included, which could lead to bias [7, 8, 18, 19, 21]. Additionally, the dataset that we used was well-managed, which included well-annotated clinicopathologic data and complete follow-up information. So, our data was more comprehensive.

To date, whether a prior cancer history affects NSCLC patients’ survivals has not been clearly elucidated. On one hand, some researchers demonstrated that prior cancers and previous treatments may have an adverse impact on the survivals of this population subset [7]. On the other hand, patients with a prior cancer might be more compliant with regular reexaminations. It is no doubt that early detection and timely management of the NSCLC could improve these patients’ survivals. After balancing the baseline differences such as tumor stages, treatment modalities and comorbidities, our results found that patients with and without prior cancer still had similar survivals. Our findings were meaningful. In the current clinical practice, the prior cancer history has prevented many patients from having access to new therapies, which might improve their survivals [9, 10]. Furthermore, exclusion of these patients could halt the pace and generalizability of clinical trials [11, 12]. Our study added to the existing body of evidence on the topic that it was reasonable for clinical trials to enroll the NSCLC patients with a prior cancer.

Our study had several limitations. First, our database lacked the information of previous cancer status and prior cancer treatments modalities and timings, which were important prognosis factors for survivals. Therefore, well-managed individual databases are warranted to validate our results. Second, only resected patients were included in this study because the data about unresected patients is unavailable. Therefore, patient selection bias might exist in our study. In addition, our results should be cautiously interpreted owing to the relatively small number of patients with a prior cancer and the retrospective design of this study. Multicenter studies with large volume are necessary to validate our results.

## Conclusions

In conclusion, our results demonstrated that resected NSCLC patients with and without a prior cancer had comparable survival rates. Herein, we proposed that it might be reasonable for clinical trials to enroll the NSCLC patients with a prior cancer.

## Supplementary Information

**Additional file 1.** 

**Additional file 2.** 

**Additional file 3.**

## Data Availability

Data from this study are available to any interested researchers upon reasonable request to the corresponding author.

## Abbreviations

NSCLC: Non-small cell lung cancer
OS: Overall survival
DFS: Disease-free survival
PSM: Propensity score matching
LASSO: Least absolute shrinkage and selection operator
CT: Computed tomography
PET: Positron emission tomography
NCCN: National Comprehensive Cancer Network
TNM: Tumor-node-metastasis
VATS: Ideo-assisted thoracic surgery
ASA: American Society of Anesthesiologists
VPI: Visceral pleural invasion
LVI: Lymphovascular invasion
BMI: Body mass index
FEV1: Forced expiratory volume in 1 s
DLCO: Diffusion capacity for carbon monoxide
NCDB: The National Cancer Database
SEER: The Surveillance, Epidemiology and End Results
ADC: Adenocarcinoma
SCC: Squamous cell carcinoma
