# Supplementary figures and images for: Is prior cancer history a hindrance for non-small cell lung cancer patients to participate in clinical trials?
Source: BMC Cancer. 2023 Feb 15;23:155. doi: 10.1186/s12885-023-10551-9 (PMC9930244; doi:10.1186/s12885-023-10551-9)

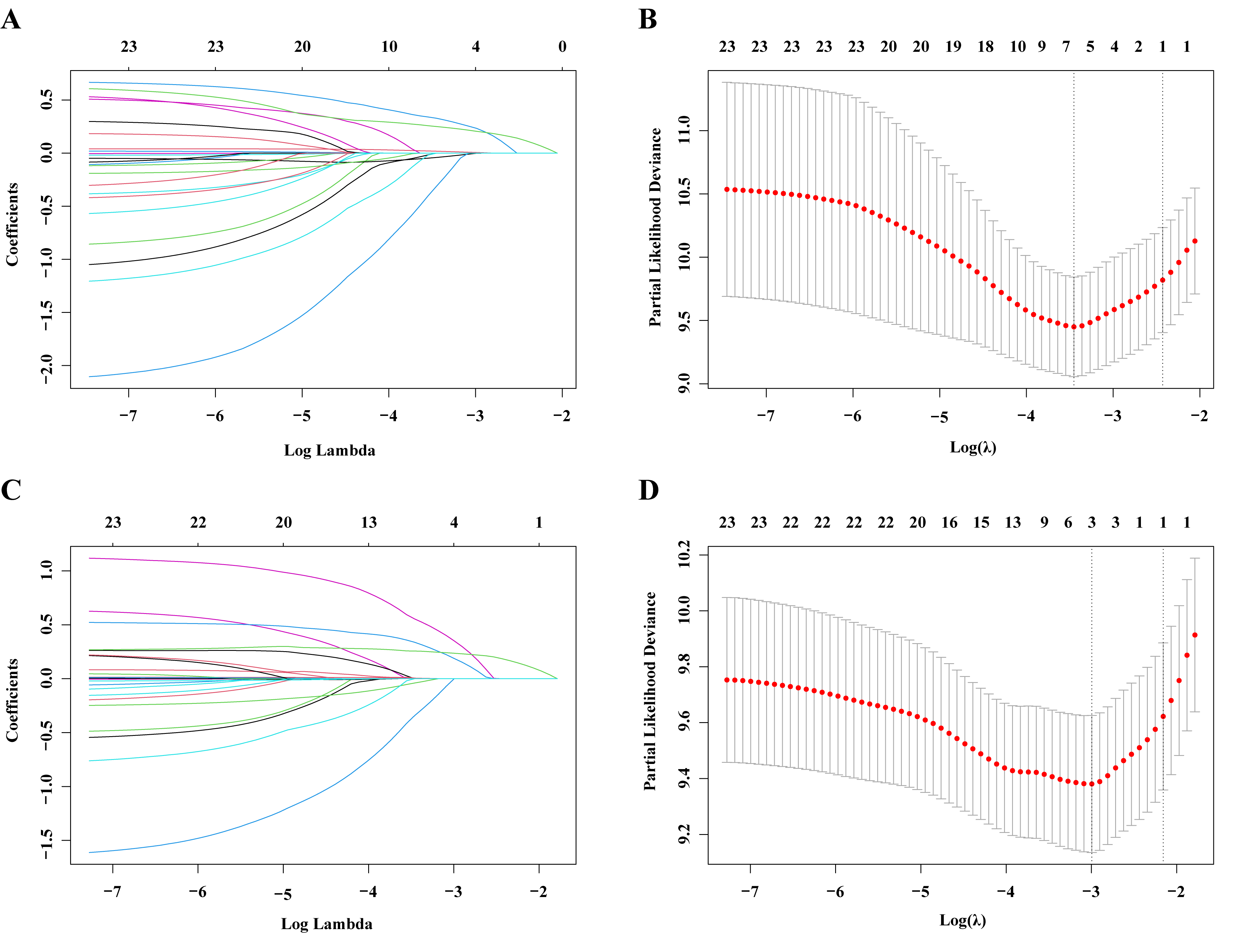

Supplement: Supplementary file 1 — Additional file 1. [file 12885_2023_10551_MOESM1_ESM.tif]

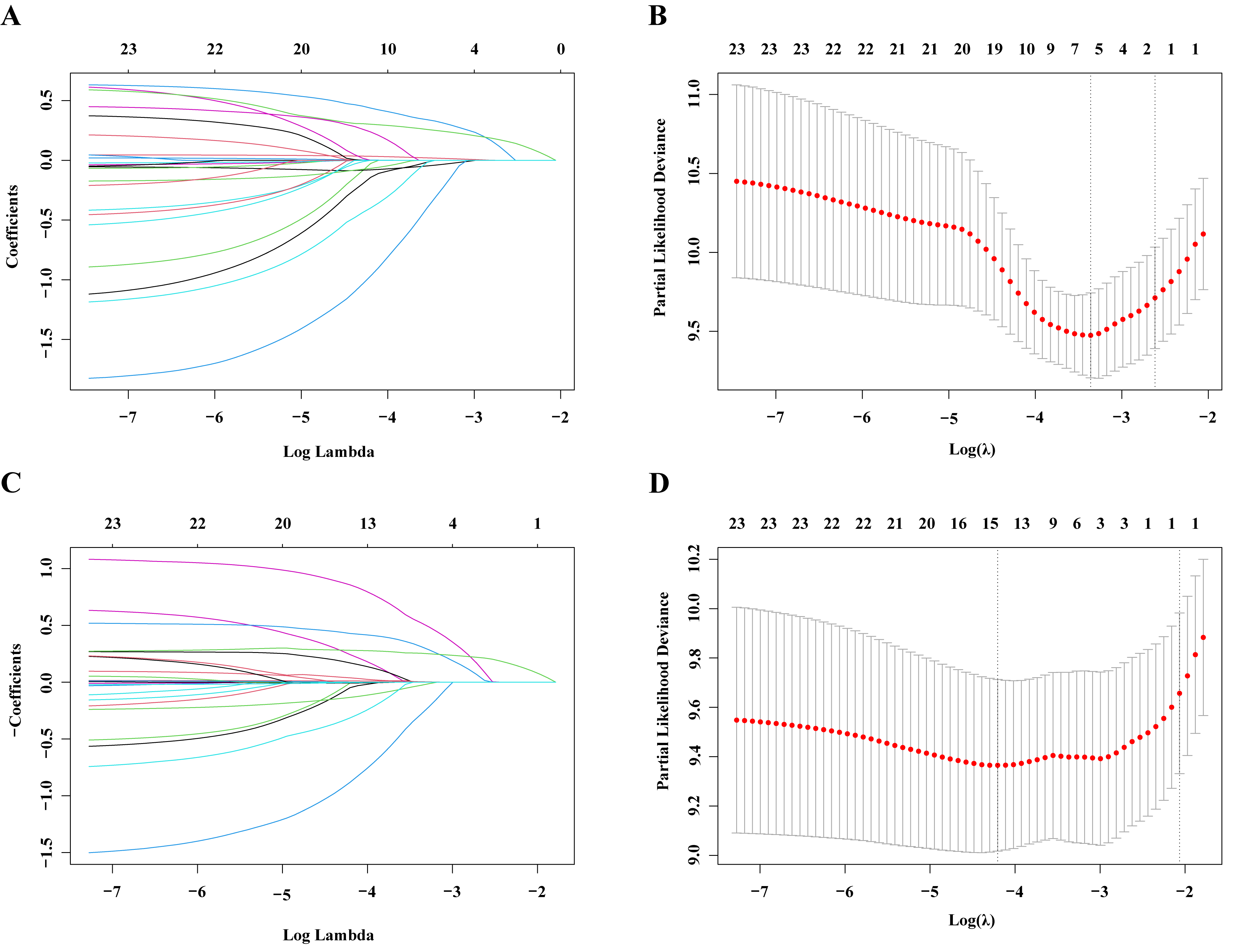

Supplement: Supplementary file 2 — Additional file 2. [file 12885_2023_10551_MOESM2_ESM.tif]
