# Supplementary material for: Is prior cancer history a hindrance for non-small cell lung cancer patients to participate in clinical trials?
Source: BMC Cancer. 2023 Feb 15;23:155. doi: 10.1186/s12885-023-10551-9 (PMC9930244; doi:10.1186/s12885-023-10551-9)
Supplement: Supplementary file 3 — Additional file 3. [file 12885_2023_10551_MOESM3_ESM.docx]

**Table S1. The baseline characteristics of the NSCLC patients with and without prior cancer after PSM**

| Characteristics | with prior cancer  (N = 338) | without prior cancer  (N = 338) | *P* |
| --- | --- | --- | --- |
| Age, years |  |  | 0.391^a^ |
| Median (range) | 65 (29-84) | 63 (31-83) |  |
| Sex |  |  | 0.408 |
| Male | 112 (33.1) | 102 (30.2) |  |
| Female | 226 (66.9) | 236 (69.8) |  |
| Smoking |  |  | 0.368 |
| Non-smoker | 252 (74.6) | 262 (77.5) |  |
| Smoker | 86 (25.4) | 76 (22.5) |  |
| Preoperative comorbidity |  |  | 0.528 |
| Without | 127 (37.6) | 135 (39.9) |  |
| With | 211 (62.4) | 203 (60.1) |  |
| BMI |  |  | 0.626^a^ |
| Median (range) | 23.7 (15.6-38.2) | 23.8 (17.0-35.9) |  |
| ASA grade |  |  | 0.914^b^ |
| 1 | 36 (10.7) | 40 (11.8) |  |
| 2 | 291 (86.1) | 282 (83.4) |  |
| 3 | 11 (3.3) | 15 (4.4) |  |
| 4 | 0 (0.0) | 1 (0.4) |  |
| Surgical approach |  |  | 1.000 |
| VATS | 315 (93.2) | 315 (93.2) |  |
| Open | 23 (6.8) | 23 (6.8) |  |
| Surgical extent |  |  | 0.811 |
| Lobectomy | 230 (68.0) | 230 (68.0) |  |
| Sublobectomy | 104 (30.8) | 102 (30.2) |  |
| Pneumonectomy | 4 (1.2) | 6 (1.8) |  |
| Histology |  |  | 0.381 |
| ADC | 282 (83.4) | 288 (85.2) |  |
| SCC | 45 (13.3) | 35 (10.4) |  |
| Other | 11 (3.3) | 15 (4.4) |  |
| VPI |  |  | 0.634 |
| Without | 271 (80.2) | 266 (78.7) |  |
| With | 67 (19.8) | 72 (21.3) |  |
| LVI |  |  | 0.454 |
| Without | 299 (88.5) | 305 (90.2) |  |
| With | 39 (11.5) | 33 (9.8) |  |
| Pathologic TNM stage |  |  | 0.273 |
| I | 269 (79.6)) | 281 (83.7) |  |
| II | 26 (7.7) | 27 (8.0) |  |
| III | 43 (12.7) | 30 (8.9) |  |
| Postoperative complication |  |  | 0.744 |
| Without | 317 (93.8) | 319 (94.4) |  |
| With | 21 (6.2) | 19 (5.6) |  |
| Adjuvant therapy |  |  | 0.376 |
| No | 270 (79.9) | 279 (82.5) |  |
| Yes | 68 (20.1) | 59 (17.5) |  |

^a^ Mann-Whitney U test

^b^ Fisher’s exact test

NSCLC, non-small cell lung cancer; PSM, propensity score matching; BMI, body mass index; TNM, tumor-node-metastasis; ASA, the American Society of Anesthesiologists; VATS, video assisted thoracic surgery; ADC, adenocarcinoma; SCC, squamous cell carcinoma; VPI, visceral pleural invasion; LVI, lymphovascular invasion

**Figure legend of supplement figures**

**Figure S1**. Prognostic factors selection for survivals using the LASSO regression model before PSM. LASSO coefficient profiles of 24 included variables against the log (Lambda) sequence for OS (A) and DFS (C). Tuning parameter (Lambda) selection in the LASSO model used 10-fold cross-validation via minimum criteria (OS: B; DFS: D). LASSO, least absolute shrinkage and selection operator; PSM, propensity score matching; OS, overall survival; DFS, disease-free survival

**Figure S2**. Prognostic factors selection for survivals using the LASSO regression model after PSM. LASSO coefficient profiles of 24 included variables against the log (Lambda) sequence for OS (A) and DFS (C). Tuning parameter (Lambda) selection in the LASSO model used 10-fold cross-validation via minimum criteria (OS: B; DFS: D). LASSO, least absolute shrinkage and selection operator; PSM, propensity score matching; OS, overall survival; DFS, disease-free survival
